# Supplementary material for: Factors Influencing the Repeated Transient Optical Droplet Vaporization Threshold and Lifetimes of Phase Change, Perfluorocarbon Nanodroplets
Source: Nanomaterials (Basel). 2023 Aug 2;13(15):2238. doi: 10.3390/nano13152238 (PMC10421047; doi:10.3390/nano13152238)
Supplement: Supplementary file 1 [file nanomaterials-13-02238-s001.zip › nanomaterials-2511399-supplementary.pdf]

Photobleaching of Epolight 3072

Epolight 3072 was dissolved in DMSO at an OD of ~1 at 1064 nm. DMSO was chosen due to the fact that it is a non-volatile organic solvent to prevent changes in the absorption spectrum due to solvent evaporation. The solution (1 mL) was placed in a glass cuvette and lased at 90 mJ/cm^2 at a PRF of 10 Hz (Vibrant, Opotek) for 400 pulses. UV-vis spectroscopy was performed at 100 pulse intervals to characterize the changes in the absorption. The spectra were processed by applying a moving average filter with a window size of three to minimize noise. Epolight 3072 appears to be resilient to photobleaching up to at least 400 pulses at 90 mJ/cm^2.

Surface wave elastography

In ultrasound elastography, shear modulus (G) can be estimated from the measured shear wave speed (c_{sh}) as:

$$G = c_{sh}^2 \rho \tag{S1}$$

where ρ is the density of the tissue, and the relationship between Young's modulus (E) and shear modulus is as follows:

$$E = 2\mathrm{G}(1 + \sigma) \tag{S2}$$

Poisson's ratios (σ) for polyacrylamide was found to be 0.5,[4] therefore the equation can be simplified and combined with equation S1 to:

$$E = 3\mathrm{G} = 3c_{sh}^2 \rho \tag{S3}$$

Furthermore, as the polyacrylamide phantom lacks acoustic scattering, the propagation speed of surface Rayleigh waves (c_{sf}) were utilized to measure the tissue elasticity. The ratio of shear wave speed to surface wave speed (c_{sh}/c_{sf}) for viscoelastic materials with σ between 0.45 and 0.499 has been shown to be $c_{sh}/c_{sf} = 1.05$.[5] As a result, surface wave speed can be related to the Young's modulus by:

$$E = 3\left(1.05\, c_{sf}\right)^2 \rho \tag{S4}$$

In this study, surface wave elastography was implemented using a Vantage 128 (Verasonics, Inc.) ultrasound imaging system equipped with a L22-14vLF linear array transducer. Waves were excited at the surface of the phantom (FIG. S2A) by an acoustic radiation force impulse (15 MHz, 1500 cycles), and ultrafast plane wave imaging (15 MHz center frequency, 15.8 kHz frame rate, 3 compounding angles) was used for data acquisition. Axial displacement was estimated from the IQ data using the Loupas 2-D autocorrelator[6]. Surface waves were excited at 5 different pushing locations (FIG. S2C) and wave speeds determined based on a time-to-peak detection. The surface wave speed in the polyacrylamide phantom was determined to be 5.51 ± 0.09 m/s and, using equation S4, the Young's modulus was calculated to be 100.26 ± 3.11 kPa.

Physical and material properties of the nanodroplets and their components

Table S1. Size and composition of Nanodroplets.

Core Material	Dye	Shell Material	Z-average size	Polydispersity index (PDI)
Perfluoropentane	Epolight 3072	Lipid	308.97	0.211
Perfluorohexane	Epolight 3072	Lipid	293.6	0.185
Perfluoroheptane	Epolight 3072	Lipid	265.13	0.248
Perfluorohexane	Epolight 3072	BSA	314.1	0.186
Perfluorohexane	Epolight 3072	Zonyl	285.8	0.167

Table S2. Critical Temperature of Cores used in Nanodroplets.

Perfluorocarbon	Chemical Formula	Critical Temperature (K)	Boiling Point (K)

Perfluoropentane	C_5F_{12}	421.9[a]	302.15
Perfluorohexane	C_6F_{14}	448.77[b]	329.15
Perfluoroheptane	C_7F_{16}	475[c]	353.15

[a]From[1]; [b] From [2]; [c] From [3].

Photobleaching of Epolight 3072

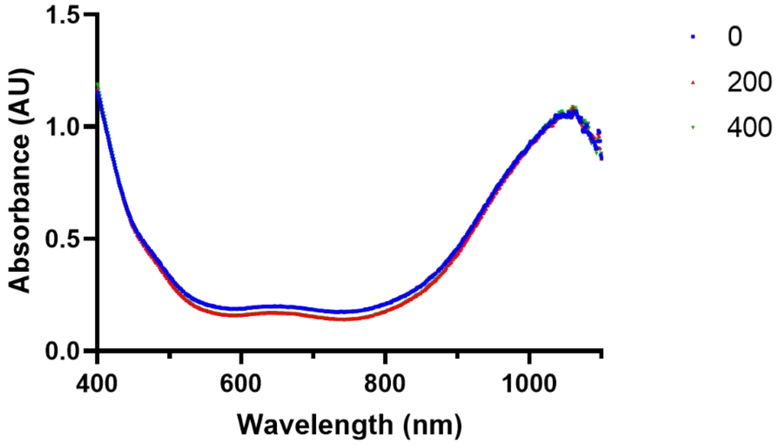

Figure S1. UV-vis spectra comparison of Epolight 3072 in DMSO before and after being lased at 1064 nm at 90 mJ/cm² for 0, 200, and 400 pulses.

Surface wave elastography

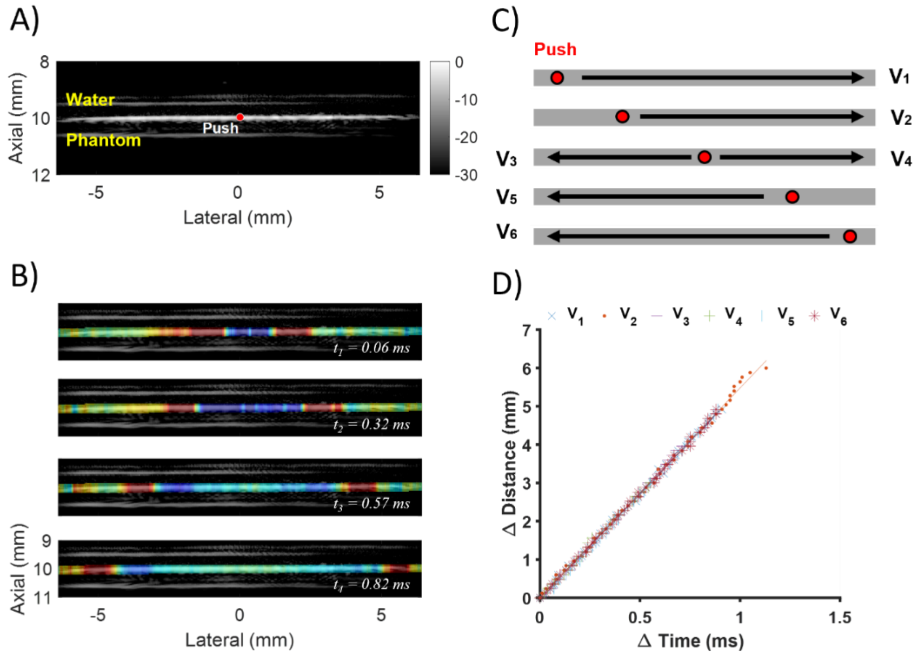

Figure S2. A) Ultrasound image of the interface between the phantom and the water along with the push location. B) The interface overlayed with multiple frames of the calculated axial displacement, depicting the propagation of surface waves over time. C) A schematic illustrating the push locations and the direction of wave speed measurement from each push. D) Wave speeds were calculated by a linear fit of the distance traveled by the peak of the wave over time. The surface wave speed in the polyacrylamide phantom was determined to be 5.51 ± 0.09 m/s and the Young's modulus was calculated to be 100.26 ± 3.11 kPa.

References

1. Ermakov, G.V.; Skripov, V.P. Saturation Line, Critical Parameters, and Attainable Superheating of the Perfluoroparaffins. *Russ. J. Phys. Chem. (Engl. Transl.)* **1967**, *41*, 39–43.
2. Mousa, A.H.N. Study of Vapor Pressure and Critical Properties of Perfluoro-n-Hexane. *J. Chem. Eng. Data* **1978**, *23*, 133–134.
3. Steele, W.V.; Chirico, R.D.; Knipmeyer, S.E.; Nguyen, A. Vapor Pressure, Heat Capacity, and Density along the Saturation Line, Measurements for Dimethyl Isophthalate, Dimethyl Carbonate, 1,3,5-Triethylbenzene, Pentafluorophenol, 4- *Tert* -Butylcatechol, α-Methylstyrene, and *N* , *N* ′-Bis(2-Hydroxyethyl)Ethylenediamine. *Journal of Chemical & Engineering Data* **1997**, *42*, 1008–1020, doi:10.1021/je970102d.
4. Boudou, T.; Ohayon, J.; Picart, C.; Pettigrew, R.I.; Tracqui, P. Nonlinear Elastic Properties of Polyacrylamide Gels: Implications for Quantification of Cellular Forces. *Biorheology* **2009**, *46*, 191–205, doi:10.3233/BIR-2009-0540.
5. Zhang, X.; Greenleaf, J.F. Estimation of Tissue's Elasticity with Surface Wave Speed. *J. Acoust. Soc. Am.* **2007**, *122*, 2522, doi:10.1121/1.2785045.
6. Loupas, T.; Powers, J.T.; Gill, R.W. An Axial Velocity Estimator for Ultrasound Blood Flow Imaging, Based on a Full Evaluation of the Doppler Equation by Means of a Two-Dimensional Autocorrelation Approach. *IEEE Trans. Ultrason., Ferroelect., Freq. Contr.* **1995**, *42*, 672–688, doi:10.1109/58.393110.
